# In vitro activity of fidaxomicin and combinations of fidaxomicin with other antibiotics against *Clostridium perfringens* strains isolated from dogs and cats

**DOI:** 10.1186/s12917-023-03801-2

**Published:** 2023-11-16

**Authors:** Sergio Álvarez-Pérez, Blanca Anega, José L. Blanco, Marta Hernández, Marta E. García

**Affiliations:** 1 Department of Animal Health, Faculty of Veterinary Medicine, Complutense University of Madrid, Madrid, Spain; 2 Laboratorio de Biología Molecular y Microbiología, Instituto Tecnológico Agrario de Castilla y León, Valladolid, Spain

**Keywords:** Antibiotic combination, Cat, *Clostridium perfringens*, Dog, Fidaxomicin

## Abstract

**Background:**

Previous studies have demonstrated that fidaxomicin, a macrocyclic lactone antibiotic used to treat recurrent *Clostridioides difficile*-associated diarrhea, also displays potent in vitro bactericidal activity against *Clostridium perfringens* strains isolated from humans. However, to date, there is no data on the susceptibility to fidaxomicin of *C. perfringens* strains of animal origin. On the other hand, although combination therapy has become popular in human and veterinary medicine, limited data are available on the effects of antibiotic combinations on *C. perfringens*. We studied the in vitro response of 21 *C. perfringens* strains obtained from dogs and cats to fidaxomicin and combinations of fidaxomicin with six other antibiotics.

**Results:**

When tested by an agar dilution method, fidaxomicin minimum inhibitory concentrations (MICs) ranged between 0.004 and 0.032 µg/ml. Moreover, the results of Etest-based combination assays revealed that the incorporation of fidaxomicin into the test medium at a concentration equivalent to half the MIC significantly increased the susceptibility of isolates to metronidazole and erythromycin in 71.4% and 61.9% of the strains, respectively, and the susceptibility to clindamycin, imipenem, levofloxacin, and vancomycin in 42.9–52.4% of the strains. In contrast, ¼ × MIC concentrations of fidaxomicin did not have any effect on levofloxacin and vancomycin MICs and only enhanced the effects of clindamycin, erythromycin, imipenem, and metronidazole in ≤ 23.8% of the tested strains.

**Conclusions:**

The results of this study demonstrate that fidaxomicin is highly effective against *C. perfringens* strains of canine and feline origin. Although fidaxomicin is currently considered a critically important antimicrobial that has not yet been licensed for veterinary use, we consider that the results reported in this paper provide useful baseline data to track the possible emergence of fidaxomicin resistant strains of *C. perfringens* in the veterinary setting.

**Supplementary Information:**

The online version contains supplementary material available at 10.1186/s12917-023-03801-2.

## Background

The Gram-positive, spore-forming, toxin-producing anaerobe *Clostridium perfringens* is a common enteropathogen of humans and diverse animals, including dogs and cats [1–3]. Previous studies have demonstrated that *C. perfringens* strains from different sources often show resistance or decreased susceptibility to diverse antibiotics, including first-line anti-anaerobic drugs such as metronidazole [4–10].

Fidaxomicin is a macrocyclic lactone antibiotic that targets RNA polymerase and has bactericidal activity against *Clostridioides difficile* (formerly *Clostridium difficile*) and other clostridia, including *C. perfringens* [11–15]. In general, most *C. perfringens* strains analyzed to date have shown low minimum inhibitory concentrations (MICs) to fidaxomicin (typically ≤ 0.004–0.06 µg/ml) [11, 12, 14]. Furthermore, fidaxomicin has multiple benefits compared to other antibiotics used to treat clostridial gastrointestinal infections, such as its good safety and tolerability profile, its low fecal binding and minimal systemic absorption, and the fact that it has minimal effect on the normal gut microbiota [11, 13, 15]. Nevertheless, the elevated acquisition cost is a major drawback of fidaxomicin (e.g., USD 3845.44 vs. USD 23.28 for a 10-day course of fidaxomicin and vancomycin, respectively [16]), even when it has been claimed that, in some contexts, fidaxomicin use might reduce total healthcare costs with respect to vancomycin or metronidazole [17–19].

Economical aspects have also precluded the use of fidaxomicin in veterinary medicine, as well as the inclusion of this antibiotic in the World Health Organization’s list of critically important antimicrobials (CIAs) for human medicine [20] and the European Union’s list of antimicrobials reserved for treatment of certain infections in humans [21]. However, given the zoonotic potential often attributed to *C. perfringens* [22], it is important to provide baseline data on the susceptibility of animal isolates of this pathogen to fidaxomicin, either alone or in combination with other antimicrobial drugs. Accordingly, in this study we analyzed the in vitro response of *C. perfringens* strains from dogs and cats to fidaxomicin and several combinations of fidaxomicin with other antibiotics.

## Results

The MICs to fidaxomicin obtained by the Clinical and Laboratory Standards Institute (CLSI) agar dilution method [23] for the *C. perfringens* strains analyzed in this study (*n* = 21) ranged from 0.004 to 0.032 µg/ml (median value: 0.008 µg/mL; Table S1). Furthermore, the fidaxomicin MIC distributions obtained for strains of different toxinotype and animal origin were overlapping: toxinotype A, 0.004 to 0.032 µg/ml (*n* = 19); toxinotype F, 0.008 to 0.016 µg/ml (*n* = 2); dog origin, 0.008 to 0.032 µg/ml (*n* = 19); and cat origin, 0.004 to 0.016 µg/ml (*n* = 4). On the other hand, the MICs to the other antibiotics used in the combination assays (see below) were as follows: clindamycin, < 0.016 to 1 µg/ml; erythromycin, 0.5 to 4 µg/ml; imipenem, 0.032 to ≥ 32 µg/ml; levofloxacin, 0.125 to 2 µg/ml; metronidazole, 4 to 64 µg/ml; and vancomycin, 0.5 to 2 µg/ml (Table S1).

Table 1 shows an overview of the results obtained in the combination assays of fidaxomicin with the aforementioned six antibiotics (see detailed results in Table S1). When tested at concentrations equivalent to half the MICs determined by the agar dilution agar (BBA + 1/2F medium, see Methods), fidaxomicin significantly enhanced the effect of the other antibiotics tested for ≥ 42.9% of the *C. perfringens* strains, with the highest frequency of significant MIC reduction being detected between fidaxomicin and metronidazole (71.4% of strains; Table 1). Moreover, the combinations of fidaxomicin with clindamycin, imipenem, erythromycin, and metronidazole, yielded variable outcomes (i.e., those instances in which different categorical results –significant increase or decrease of the MIC values, or non-significant MIC variation– were observed in two replicates of the combination assay) for 9.5 to 19% of strains (Table 1). In contrast, significant activity enhancement was remarkably less frequent for all antibiotic combinations tested when fidaxomicin was present in BBA at a quarter of the MIC of the tested strains (BBA + 1/4F medium, see Methods; Table 1). Significant activity reduction (i.e., MIC increase) could not be confirmed for any antibiotic combination tested; nevertheless, two strains that yielded a variable result (namely, G/05P1 and M/14P3) showed a > 2-fold dilution increase in the MIC to clindamycin in one of the test replicates in BBA + 1/4F. Finally, it was found that for all antibiotic pairs except fidaxomicin-clindamycin the frequency of each outcome of the combination assay significantly depended on the concentration of fidaxomicin included in the test medium (*P* = 0.331 for fidaxomicin-clindamycin; *P* < 0.001 for all other antibiotic combinations).

**Table 1:** Overview of the results of the assays testing the interaction of fidaxomicin with other antibiotics against *Clostridium perfringens* isolates from dogs and cats (*n* = 21)

Test medium^*a*^	Combined antibiotic^*b*^	Outcome of the interaction^*c*^		
		Significant activity enhancement	Non-significant MIC variation	Variable result
BBA + 1/2F	Clindamycin	10 (47.6%)	9 (42.9%)	2 (9.5%)
	Erythromycin	13 (61.9%)	5 (23.8%)	3 (14.3%)
	Imipenem	9 (42.9%)	8 (38.1%)	4 (19%)
	Levofloxacin	10 (47.6%)	11 (52.4%)	0 (0%)
	Metronidazole	15 (71.4%)	3 (14.3%)	3 (14.3%)
	Vancomycin	11 (52.4%)	10 (47.6%)	0 (0%)
BBA + 1/4F	Clindamycin	5 (23.8%)	12 (57.1%)	4 (19%)
	Erythromycin	1 (4.8%)	19 (90.5%)	1 (4.8%)
	Imipenem	1 (4.8%)	20 (95.2%)	0 (0%)
	Levofloxacin	0 (0%)	21 (100%)	0 (0%)
	Metronidazole	4 (19%)	16 (76.2%)	1 (4.8%)
	Vancomycin	0 (0%)	21 (100%)	0 (0%)

^*a*^ BBA + 1/2F and BBA + 1/4F refer to Brucella blood agar with hemin and vitamin K (BBA) supplemented with fidaxomicin at ½ × and ¼ × the minimum inhibitory concentration (MIC) determined by the CLSI agar dilution method, respectively

^*b*^ Etest strips placed on BBA + 1/2F and BBA + 1/4F in the combination assay

^*c*^ Frequency (and percentage) of each outcome. Significant activity enhancement: ≥3 two-fold reduction in the MIC when compared to control plates containing no fidaxomicin (BBA + 0 F); non-significant MIC variation: ≤2 two-fold MIC change compared to BBA + 0 F; variable result: cases in which different categorical results (significant activity enhancement, significant activity reduction, or non-significant MIC variation) were observed in two replicates of the combination assay

## Discussion

Although fidaxomicin is not currently used in veterinary medicine, given the widespread occurrence of antibiotic-resistant *C. perfringens* in animals and the environment (see, e.g. [4–6, 9]) and the ‘One Health’ approach proposed for the study of other clostridia of similar ecology (e.g., *C. difficile* [24, 25] and *Clostridium botulinum* [26]), animal strains of *C. perfringens* should also be tested for in vitro susceptibility to fidaxomicin. However, to our knowledge, no other previous studies have addressed this issue. To fill in this research gap, we analyzed the in vitro effect of fidaxomicin against 21 *C. perfringens* strains of canine and feline origin. Our results showed than, when tested by the CLSI agar dilution method [23], fidaxomicin MICs were low (0.004–0.032 µg/ml), which agrees with the results of previous studies testing *C. perfringens* strains of human origin and the potent bactericidal activity that this antibiotic has against *C. difficile* and other clostridia *sensu lato*, e.g., *Clostridium butyricum*, *Clostridium paraputrificum*, *Paraclostridium bifermentans* (formerly *Clostridium bifermentans*), and *Terrisporobacter glycolicus* (formerly *Clostridium glycolicum*) [11, 12, 14]. Similarly, unpublished data from our research group indicates that strains isolated from intensively-raised pigs and Iberian pigs also display low fidaxomicin MICs (typically ≤ 0.032 µg/ml; García M.E. et al., unpublished data). In contrast, other species such as *Clostridium innocuum*, *Enterocloster bolteae* (formerly *Clostridium bolteae*), *Hungatella hathewayi* (formerly *Clostridium hathewayi*), and *Thomasclavelia ramosa* (formerly *Clostridium ramosum*) seem to be intrinsically resistant to fidaxomicin, and a human isolate of *C. perfringens* with a MIC value of 64 µg/ml has been found in Japan [12, 14].

On the other hand, antibiotic combination therapy has become popular in human and veterinary medicine as a strategy to enhance the efficacy of antibiotic treatments against diverse bacterial pathogens while reducing the undesirable side effects of such treatments and slowing down the development of resistance [27–29]. To our knowledge, the susceptibility of *C. perfringens* to antibiotic combinations has never been assessed, even when, for example, some combinations of metronidazole with vancomycin, macrolides, quinolones, beta-lactams, and/or rifaximin are often used or have been tested in clinical trials to treat a variety of digestive disorders in humans and pets [30–34]. In the present study, we analyzed the in vitro response of *C. perfringens* to combinations of fidaxomicin with other six antibiotics and found that the outcome of the interaction assays depended on the combined antibiotics, the concentration of fidaxomicin in the test medium, and the strain. In particular, the incorporation of fidaxomicin into the test medium at half the MIC determined by the agar dilution method significantly enhanced the activity (i.e., decreased the MIC values) of clindamycin, erythromycin, levofloxacin, imipenem, metronidazole, and vancomycin in > 40% of the tested strains. In contrast, concentrations of fidaxomicin equivalent to a quarter of the MIC resulted in non-significant variation oflevofloxacin or vancomycin MICs and reduced the frequency of significant activity enhancement of clindamycin, erythromycin, imipenem, and metronidazole. Similar strain-, compound-, and/or concentration-dependent antibiotic combination effects have been reported for other bacteria (e.g., carbapenemase-producing enterobacteria [35], methicillin-resistant *Staphylococcus aureus* [36], and vancomycin-resistant enterococci [37]), which makes it difficult to generalize about the effects of a particular antibiotic combinations against a given pathogen and highlights the need for baseline data such as those reported here.

A limitation of this study is that the results of combination assays are generally interpreted using the fractional inhibitory concentration index (FICI; which is calculated using the following formula: FICI = MIC_AB_/MIC_A_ + MIC_BA_/MIC_B_, where MIC_A_ and MIC_B_ are the MICs of drugs A and B when acting alone and MIC_AB_ and MIC_BA_ are the MICs of drugs A and B when acting in combination, respectively) and by interpreting the possible results in terms of ‘synergy’ (FICI ≤ 0.5), ‘antagonism’ (FICI > 4), and ‘indifference’ or ‘no interaction’ (FICI > 0.5–4) [38, 39]. Alternative definitions of these concepts have been proposed by other authors for those cases where one of the antibiotics is included in an agar medium at a fixed concentration (i.e., fidaxomicin in the present study) but there is a concentration gradient of the other antibiotic used in the combination assay (e.g., created by using Etest strips) [40, 41]. However, in absence of a clear consensus for these alternative definitions of synergy and antagonism, we have interpreted our results in terms of significant activity enhancement, significant activity reduction, or non-significant MIC variation. Furthermore, the limited number of *C. perfringens* strains of toxinotype F and cat origin tested in this study (two and four, respectively), preclude a reliable analysis of toxinotype- and host-based differences in fidaxomicin susceptibility and combination effects. Additionally, although the strains included in this study were genetically diverse (see Methods), we acknowledge that our limited selection of strains might not represent the whole intra-species genetic diversity of *C. perfringens* and, therefore, future studies should confirm if our conclusions are applicable to strains of other genetic backgrounds. Despite these limitations of our study and the fact that fidaxomicin is currently considered a CIA that should be reserved for the treatment of certain infections in humans and, accordingly, that it has not yet been licensed for veterinary use, we consider that the results here reported provide useful baseline data to track the possible emergence of fidaxomicin resistant strains of *C. perfringens* in the veterinary setting. In any case, as already done for other CIAs, the eventual use of fidaxomicin in the treatment of animal infections should follow the guidelines and recommendations of international, national, and/or regional agencies for medicinal products and animal health (see, for example, Refs. [42–44]).

## Conclusion

Although the use of fidaxomicin is currently restricted to human medicine, the results of this study demonstrate that this antibiotic is also highly effective against *C. perfringens* toxinotype A and F strains of canine and feline origin. Moreover, our results reveal that the occurrence of in vitro combination effects between fidaxomicin and other antibiotics against *C. perfringens* is compound-, concentration-, and strain-dependent. Future research should clarify if these conclusions can also be applied to other toxinotypes of *C. perfringens* and/or strains from other sources and genetic backgrounds.

## Methods

### Strains

A total of 21 *C. perfringens* strains obtained from fecal samples of dogs (*n* = 17 strains) and cats (*n* = 4) attended between 24 and 2015 and 1 December 2015 at different primary care veterinary clinics located in the Madrid region, Spain (see details in Álvarez-Pérez et al. [5, 45]) were included in the present study. The selection of strains was mainly done based on their MIC values to fidaxomicin and other antibiotics (see Results), so as to have a representation of strains with different antibiotic susceptibility profiles in the combination assays described below. All strains had been primarily recovered in Columbia blood agar (bioMérieux, Marcy l’Etoile, France) and stored at − 70 °C as cell suspensions in brain heart infusion broth (BHI; Pronadisa, Madrid, Spain) supplemented with 25% glycerol (Panreac, Barcelona, Spain). PCR toxinotyping revealed that 19 of the studied strains, including 15 strains from dogs and the four strains from cats, belonged to toxinotype A (they only had the *cpa* gene encoding for *C. perfringens* alpha toxin), whereas the other two strains of canine origin should be classified as toxinotype F (besides *cpa*, these strains also had the *cpe* gene enconding *C. perfringens* enterotoxin) [5, 45, 46]. Furthermore, all studied strains belonged to different amplified fragment length polymorphism genotypes [5, 45].

### Fidaxomicin susceptibility testing

In vitro susceptibility to fidaxomicin (0.001 to 0.125 µg/ml; Sigma-Aldrich, Madrid, Spain) was determined by the agar dilution method, which was performed by following the CLSI guidelines for anaerobes [23], using dimethyl sulfoxide (DMSO; Sigma-Aldrich) as the antibiotic solvent and Brucella blood agar with hemin (5 µg/ml; Sigma-Aldrich), vitamin K (1 µg/ml; Sigma-Aldrich), and 5% v/v of defibrinated sheep blood (Oxoid Ltd., Basingstoke, UK) (BBA) as culture medium,. All strains were tested at least twice on different days, and drug-free plates were included as growth controls.

### Combination assays

Analysis of the response of *C. perfringens* strains to combinations of fidaxomicin with other six antibiotics, namely clindamycin, erythromycin, imipenem, levofloxacin, metronidazole, and vancomycin was performed by an Etest-based method. Briefly, based on the MIC results determined by the agar dilution method (see above), two series of 90-mm-diameter plates containing 20 ml of BBA supplemented with fidaxomicin at ½ × MIC and ¼ × MIC concentrations (hereafter referred to as BBA + 1/2F, BBA + 1/4F, respectively) were prepared. Additionally, plates containing 20 ml of BBA plus 1% v/v of DMSO but no fidaxomicin (BBA + 0 F) were used as controls. Assay plates were immediately used or stored at 4 °C and used within 24 h.

Cotton-tipped sterile swabs (Aptaca Spa., Canelli, Italy) were dipped in cell suspensions (McFarland standard of 1, prepared in BHI from 2 to 3-day old cultures), which were spread onto the surface of the BBA + 1/2F, BBA + 1/4F, and BBA + 0 F plates. Clindamycin, erythromycin, imipenem, levofloxacin, metronidazole, and vancomycin Etest strips (bioMérieux) were laid on the surface of inoculated plates. All plates were incubated under strictly anaerobic conditions (< 0.1% of oxygen after 2.5 h; GENbox anaer, bioMérieux) at 37 °C and read after 48 h. The effect of each antibiotic combination on each *C. perfringens* strain was tested twice on different days. The drug concentration shown on the Etest strip at the outer border of the elliptical inhibition halo was recorded as the MIC. High off-scale values were converted to the next highest concentration, whereas low off-scale MICs were left unchanged. A MIC reduction or increase of at least three two-fold dilutions in the presence of fidaxomicin in both test replicates was regarded as proof of significant activity enhancement or significant activity reduction, respectively, and ≤ 2 two-fold MIC changes were interpreted as non-significant MIC variation.

### Data analysis

Statistical analysis of results was performed using R v.4.2.2. The Fisher’s exact test for count data with simulated *P*-value (two-sided, based on 10^4^ replicates) was used for the analysis of categorical data where appropriate. *P*-values < 0.05 were considered statistically significant.

### Electronic supplementary material

Below is the link to the electronic supplementary material.

Supplementary Material 1

## Data Availability

The relevant datasets supporting the conclusions of this article are included within the article or in the supplementary materials.

## Abbreviations

BBA: Brucella blood agar with hemin and vitamin K
BBA + 0 F: BBA with no fidaxomicin
BBA + 1/2F: BBA supplemented with fidaxomicin at half the MIC
BBA + 1/4F: BBA supplemented with fidaxomicin at a quarter the MIC
BHI: brain heart infusion broth
CIA: critically important antimicrobial
CLSI: Clinical Laboratory and Standards Institute
DMSO: dimethyl sulfoxide
MIC: minimum inhibitory concentration
