# Supplementary material for: In vitro activity of fidaxomicin and combinations of fidaxomicin with other antibiotics against Clostridium perfringens strains isolated from dogs and cats
Source: BMC Vet Res. 2023 Nov 16;19:238. doi: 10.1186/s12917-023-03801-2 (PMC10652485; doi:10.1186/s12917-023-03801-2)
Supplement: Supplementary file 1 — Supplementary Material 1 [file 12917_2023_3801_MOESM1_ESM.docx]

**Table S1.** Detailed results of the Etest-based assays testing the interaction of fidaxomicin with other antibiotics against *Clostridium perfringens* isolates from dogs and cats (*n* = 21).

| **Strain (fidaxomicin MIC, µg/ml)*^a^*** | **Origin (toxin type)** | **Test plate*^b^*** | **MIC results (1^st^ replicate)*^c^*** | | | | | |  | **MIC results (2^nd^ replicate)*^c^*** | | | | | |
| --- | --- | --- | --- | --- | --- | --- | --- | --- | --- | --- | --- | --- | --- | --- | --- |
|  |  |  | **CLI** | **ERY** | **IPM** | **LVX** | **MTZ** | **VAN** |  | **CLI** | **ERY** | **IPM** | **LVX** | **MTZ** | **VAN** |
| A/02P2 (0.008) | Dog (A) | BBA+0F | 0.25 | 1 | 0.125 | 0.25 | 64 | 0.5 |  | 1 | 2 | 0.125 | 0.5 | 16 | 1 |
|  |  | BBA+½F | 0.032 (AE) | 0.25 | 0.032 | 0.25 | 2 (AE) | 0.5 |  | 0.032 (AE) | 0.5 | 0.064 | 0.25 | 4 | 1 |
|  |  | BBA+¼F | 0.125 | 2 | 0.5 | 0.25 | 32 | 0.5 |  | 0.5 | 2 | 0.5 | 0.5 | 16 | 1 |
| A/02P3 (0.008) | Dog (F) | BBA+0F | 0.25 | 0.5 | 0.064 | 0.125 | 8 | 0.5 |  | 1 | 2 | 0.25 | 0.25 | 16 | 0.5 |
|  |  | BBA+½F | <0.016 (AE) | 1 | 0.064 | 0.25 | 0.5 (AE) | 0.5 |  | <0.016 (AE) | 0.5 | <0.016 (AE) | 0.25 | 0.5 (AE) | 0.5 |
|  |  | BBA+¼F | <0.016 (AE) | 0.5 | 0.064 | 0.25 | 8 | 0.5 |  | <0.016 (AE) | 2 | 0.125 | 0.25 | 16 | 0.5 |
| B/14P1 (0.008) | Cat (A) | BBA+0F | 0.25 | 0.5 | 0.064 | 0.5 | 16 | 1 |  | 0.125 | 1 | 0.064 | 0.25 | 16 | 0.5 |
|  |  | BBA+½F | <0.016 (AE) | <0.016 (AE) | <0.002 (AE) | <0.002 (AE) | <0.016 (AE) | <0.016 (AE) |  | <0.016 (AE) | <0.016 (AE) | <0.002 (AE) | <0.002 (AE) | <0.016 (AE) | <0.016 (AE) |
|  |  | BBA+¼F | <0.016 (AE) | 0.5 | 0.125 | 0.25 | 1 (AE) | 0.5 |  | <0.016 (AE) | 0.25 | 0.064 | 0.25 | 0.5 (AE) | 0.5 |
| G/05P1 (0.016) | Dog (A) | BBA+0F | 0.064 | 2 | 0.25 | 0.5 | 64 | 1 |  | <0.016 | 2 | 0.064 | 0.5 | 16 | 1 |
|  |  | BBA+½F | 0.064 | <0.016 (AE) | <0.002 (AE) | <0.002 (AE) | <0.016 (AE) | <0.016 (AE) |  | <0.016 | <0.016 (AE) | 0.004 (AE) | <0.002 (AE) | <0.016 (AE) | <0.016 (AE) |
|  |  | BBA+¼F | 0.125 | 2 | 0.25 | 0.5 | <0.016 (AE) | 1 |  | 0.5 (AR) | 2 | 0.064 | 0.5 | <0.016 (AE) | 0.5 |
| G/06P1 (0.016) | Cat (A) | BBA+0F | 0.032 | 2 | 0.064 | 0.5 | 16 | 0.5 |  | 0.064 | 1 | 0.064 | 0.25 | 8 | 0.5 |
|  |  | BBA+½F | <0.016 | <0.016 (AE) | <0.002 (AE) | <0.002 (AE) | <0.016 (AE) | <0.016 (AE) |  | <0.016 | <0.016 (AE) | <0.002 (AE) | <0.002 (AE) | <0.016 (AE) | <0.016 (AE) |
|  |  | BBA+¼F | 0.032 | 2 | 0.064 | 0.5 | 16 | 0.5 |  | 0.125 | 2 | 0.064 | 0.5 | 8 | 1 |
| H/03P2 (0.016) | Dog (A) | BBA+0F | 0.25 | 2 | 32 | 0.5 | 16 | 1 |  | 0.125 | 4 | ≥32 | 0.5 | 8 | 1 |
|  |  | BBA+½F | <0.016 (AE) | <0.016 (AE) | <0.002 (AE) | <0.002 (AE) | <0.016 (AE) | <0.016 (AE) |  | <0.016 (AE) | <0.016 (AE) | <0.002 (AE) | <0.002 (AE) | <0.016 (AE) | <0.016 (AE) |
|  |  | BBA+¼F | 0.5 | 1 | 32 | 0.5 | 16 | 1 |  | 0.125 | 2 | ≥32 | 0.5 | 8 | 1 |
| H/03P3 (0.008) | Dog (A) | BBA+0F | 0.25 | 1 | 0.064 | 1 | 8 | 0.5 |  | 0.25 | 1 | 0.125 | 2 | 8 | 0.5 |
|  |  | BBA+½F | <0.016 (AE) | 0.5 | 0.064 | 2 | 1 (AE) | 0.5 |  | <0.016 (AE) | 0.25 | 0.032 | 4 | 0.5 (AE) | 0.5 |
|  |  | BBA+¼F | <0.016 (AE) | 2 | 0.064 | 2 | 8 | 0.5 |  | <0.016 (AE) | 2 | 0.125 | 2 | 8 | 1 |
| H/05P1 (0.008) | Dog (A) | BBA+0F | 0.25 | 2 | ≥32 | 0.25 | 8 | 0.5 |  | 0.5 | 2 | ≥32 | 0.5 | 8 | 0.5 |
|  |  | BBA+½F | <0.016 (AE) | <0.016 (AE) | <0.002 (AE) | <0.002 (AE) | <0.016 (AE) | 0.032 (AE) |  | <0.016 (AE) | <0.016 (AE) | <0.002 (AE) | <0.002 (AE) | <0.016 (AE) | <0.016 (AE) |
|  |  | BBA+¼F | 0.064 | 1 | ≥32 | 0.25 | 8 | 1 |  | <0.016 (AE) | 4 | ≥32 | 0.5 | 16 | 1 |
| J/01P1 (0.032) | Dog (A) | BBA+0F | 0.5 | 2 | 0.064 | 0.5 | 32 | 0.5 |  | 0.25 | 2 | 0.064 | 0.5 | 16 | 2 |
|  |  | BBA+½F | <0.016 (AE) | <0.016 (AE) | <0.002 (AE) | <0.002 (AE) | <0.016 (AE) | <0.016 (AE) |  | <0.016 (AE) | <0.016 (AE) | <0.002 (AE) | <0.002 (AE) | <0.016 (AE) | <0.016 (AE) |
|  |  | BBA+¼F | 0.032 (AE) | 2 | 0.064 | 0.5 | 16 | 1 |  | 0.032 (AE) | 1 | 0.032 | 0.5 | 4 | 1 |
| K/02P1 (0.008) | Dog (A) | BBA+0F | 0.064 | 2 | 0.25 | 0.5 | 32 | 0.5 |  | 0.064 | 2 | 0.064 | 0.5 | 8 | 1 |
|  |  | BBA+½F | <0.016 | <0.016 (AE) | <0.002 (AE) | <0.002 (AE) | <0.016 (AE) | <0.016 (AE) |  | <0.016 | <0.016 (AE) | <0.002 (AE) | <0.002 (AE) | <0.016 (AE) | <0.016 (AE) |
|  |  | BBA+¼F | <0.016 | 2 | <0.002 (AE) | 0.5 | 16 | 0.5 |  | 0.032 | 1 | <0.002 (AE) | 0.25 | 4 | 0.5 |
| M/06P1 (0.008) | Dog (A) | BBA+0F | <0.016 | 2 | 0.064 | 0.25 | 32 | 0.5 |  | 0.064 | 4 | 0.064 | 0.5 | 64 | 2 |
|  |  | BBA+½F | <0.016 | 1 | 0.032 | 0.25 | <0.016 (AE) | 0.5 |  | <0.016 | 1 | 0.016 | 0.5 | <0.016 (AE) | 2 |
|  |  | BBA+¼F | <0.016 | 2 | 0.064 | 0.5 | 32 | 1 |  | 0.064 | 4 | 0.064 | 1 | 8 (AE) | 2 |
| M/06P2 (0.008) | Dog (A) | BBA+0F | <0.016 | 1 | 0.064 | 0.5 | 8 | 1 |  | 0.064 | 2 | 0.064 | 0.5 | 16 | 1 |
|  |  | BBA+½F | <0.016 | 0.25 | 0.064 | 0.25 | 0.064 (AE) | 0.5 |  | <0.016 | 0.25 (AE) | 0.016 | 0.125 | 0.25 (AE) | 0.5 |
|  |  | BBA+¼F | <0.016 | 2 | 0.016 | 0.25 | 8 | 1 |  | 0.064 | 2 | 0.064 | 0.5 | 16 | 1 |
| M/08P1 (0.008) | Dog (A) | BBA+0F | 1 | 4 | 0.032 | 0.125 | 4 | 0.5 |  | 0.5 | 2 | 0.064 | 0.5 | 8 | 1 |
|  |  | BBA+½F | <0.016 (AE) | <0.016 (AE) | 0.032 | 0.25 | 0.25 (AE) | <0.016 (AE) |  | <0.016 (AE) | <0.016 (AE) | 0.032 | 0.125 | 0.125 (AE) | <0.016 (AE) |
|  |  | BBA+¼F | <0.016 (AE) | <0.016 (AE) | 0.032 | 0.25 | 2 | 1 |  | <0.016 (AE) | <0.016 (AE) | 0.064 | 0.25 | 8 | 1 |
| M/08P2 (0.008) | Dog (A) | BBA+0F | 0.25 | 2 | 0.064 | 0.5 | 8 | 0.5 |  | 0.5 | 2 | 0.125 | 0.5 | 16 | 0.5 |
|  |  | BBA+½F | <0.016 (AE) | 1 | 0.032 | 0.5 | 8 | 1 |  | <0.016 (AE) | 0.5 | 0.032 | 1 | 2 (AE) | 1 |
|  |  | BBA+¼F | 0.5 | 2 | 0.064 | 0.5 | 16 | 0.5 |  | 0.5 | 4 | 0.064 | 0.5 | 16 | 1 |
| M/08P3 (0.008) | Dog (A) | BBA+0F | 0.5 | 4 | 0.125 | 0.5 | 16 | 1 |  | 1 | 2 | 0.064 | 0.5 | 16 | 1 |
|  |  | BBA+½F | <0.016 (AE) | 0.25 (AE) | 0.016 (AE) | 0.5 | 0.25 (AE) | 0.5 |  | <0.016 (AE) | 0.5 | 0.016 | 0.5 | 1 (AE) | 1 |
|  |  | BBA+¼F | 0.5 | 4 | 0.064 | 0.5 | 16 | 1 |  | 0.25 | 4 | 0.064 | 0.5 | 16 | 1 |
| M/13P1 (0.004) | Cat (A) | BBA+0F | 0.25 | 4 | 0.125 | 0.25 | 16 | 1 |  | 0.064 | 1 | 0.064 | 0.25 | 8 | 1 |
|  |  | BBA+½F | 0.032 (AE) | 0.5 (AE) | 0.064 | 0.25 | 8 | 1 |  | 0.032 | 0.125 (AE) | 0.032 | 0.25 | 8 | 1 |
|  |  | BBA+¼F | 0.25 | 4 | 0.064 | 0.25 | 8 | 1 |  | 0.064 | 2 | 0.064 | 0.25 | 8 | 1 |
| M/13P2 (0.008) | Cat (A) | BBA+0F | 0.064 | 2 | 0.064 | 0.25 | 4 | 0.5 |  | 0.032 | 2 | 0.125 | 0.25 | 16 | 1 |
|  |  | BBA+½F | <0.016 | 0.032 (AE) | 0.016 | 0.016 (AE) | 2 | 0.5 |  | <0.016 | 0.064 (AE) | 0.008 (AE) | 0.032 (AE) | 1 (AE) | 0.25 |
|  |  | BBA+¼F | <0.016 | 1 | 0.064 | 0.25 | 8 | 1 |  | <0.016 | 2 | 0.125 | 0.25 | 16 | 1 |
| M/14P3 (0.016) | Dog (F) | BBA+0F | 0.064 | 2 | 0.064 | 0.25 | 16 | 0.5 |  | <0.016 | 1 | 0.064 | 0.25 | 4 | 0.5 |
|  |  | BBA+½F | <0.016 | <0.016 (AE) | 0.064 | 0.25 | 4 | 0.5 |  | <0.016 | <0.016 (AE) | 0.032 | 0.125 | 2 | 0.125 |
|  |  | BBA+¼F | 0.064 | 1 | 0.125 | 0.5 | 8 | 0.5 |  | 0.25 (AR) | 2 | 0.125 | 0.25 | 16 | 0.5 |
| M/26P1 (0.008) | Dog (A) | BBA+0F | <0.016 | 1 | 0.064 | 0.25 | 16 | 0.5 |  | 0.064 | 1 | 0.064 | 0.25 | 16 | 0.5 |
|  |  | BBA+½F | <0.016 | <0.016 (AE) | <0.002 (AE) | <0.002 (AE) | <0.016 (AE) | <0.016 (AE) |  | <0.016 | <0.016 (AE) | <0.002 (AE) | <0.002 (AE) | <0.016 (AE) | <0.016 (AE) |
|  |  | BBA+¼F | <0.016 | 1 | 0.064 | 0.25 | 1 (AE) | 1 |  | <0.016 | 0.25 | 0.032 | 0.064 | 1 (AE) | 0.5 |
| M/26P2 (0.008) | Dog (A) | BBA+0F | <0.016 | 1 | 0.064 | 0.25 | 8 | 0.5 |  | <0.016 | 2 | 0.125 | 0.25 | 16 | 1 |
|  |  | BBA+½F | <0.016 | 0.25 | 0.064 | 0.125 | <0.016 (AE) | 0.5 |  | <0.016 | 0.064 (AE) | 0.016 (AE) | 0.125 | <0.016 (AE) | 0.25 |
|  |  | BBA+¼F | <0.016 | 1 | 0.064 | 0.125 | 8 | 1 |  | <0.016 | 4 | 0.125 | 0.25 | 16 | 1 |
| O/11P1 (0.032) | Dog (A) | BBA+0F | 0.032 | 2 | 0.032 | 0.25 | 8 | 0.5 |  | 0.125 | 2 | 0.064 | 0.25 | 8 | 0.5 |
|  |  | BBA+½F | <0.016 | <0.016 (AE) | <0.002 (AE) | <0.002 (AE) | <0.016 (AE) | <0.016 (AE) |  | <0.016 (AE) | <0.016 (AE) | <0.002 (AE) | <0.002 (AE) | <0.016 (AE) | <0.016 (AE) |
|  |  | BBA+¼F | 0.032 | 0.25 (AE) | 0.016 | 0.125 | 0.5 (AE) | 0.5 |  | <0.016 (AE) | 1 | 0.032 | 0.25 | 1 (AE) | 0.5 |

*^a^* Fidaxomicin minimum inhibitory concentration (MIC) values were determined by the CLSI agar dilution method.

*^b^* BBA+0F: Brucella blood agar with hemin and vitamin K (BBA) containing no fidaxomicin (i.e., control plates); BBA+1/2F: BBA containing fidaxomicin at a concentration equivalent to half the MIC determined by agar dilution; BBA+1/4F: BBA containing fidaxomicin at a quarter of the MIC determined by agar dilution.

*^c^* MICs (in µg/ml) determined by the Etest method for the antibiotics combined with fidaxomicin: CLI, clindamycin; ERY, erythromycin; IPM, imipenem; LVX, levofloxacin; MTZ, metronidazole; VAN, vancomycin. AE: significant activity enhancement (i.e., ≥3 two-fold reduction in the MIC when compared to BBA+0F); AR: significant activity reduction (i.e., ≥3 two-fold increase in the MIC when compared to BBA+0F).
